# Use of gold nanoparticle-silibinin conjugates: A novel approach against lung cancer cells

**DOI:** 10.3389/fchem.2022.1018759

**Published:** 2022-10-13

**Authors:** Rangnath Ravi, Md. Zeyaullah, Shubhrima Ghosh, Mohiuddin Khan Warsi, Renu Baweja, Abdullah M. AlShahrani, Abhijeet Mishra, Razi Ahmad

**Affiliations:** ^1^ Department of Chemistry, Shivaji College, University of Delhi, New Delhi, India; ^2^ Department of Basic Medical Science, College of Applied Medical Sciences, King Khalid University (KKU), Khamis Mushayt Campus, Abha, Saudi Arabia; ^3^ Trinity Translational Medicine Institute, Trinity College Dublin, Dublin, Ireland; ^4^ Department of Biochemistry, College of Science, University of Jeddah, Jeddah, Saudi Arabia; ^5^ Department of Biochemistry, Shivaji College, University of Delhi, New Delhi, India; ^6^ Department of Chemistry, Indian Institute of Technology Delhi, New Delhi, India; ^7^ Quality and Research Department, Anantaa GSK Innovations Pvt Ltd., DLF Industrial Area, Faridabad, India

**Keywords:** gold nanoparticles, silibinin, lung cancer, phytochemicals, cancer therapy

## Abstract

Lung cancer presents one of the most challenging carcinomas with meager 5-year survival rates (less than 20%), high metastasis and high recurrence due to chemo- and radio- resistance. An alternative or complementation to existing prognosis modalities is the use of phytochemicals such as silibinin, which targets essential cytokines, angiogenic factors and transcription factors for a profound anti-tumor effect. However, the problems of low solubility in an aqueous physiological environment, poor penetration, high metabolism and rapid systemic clearance limit the therapeutic use of silibinin. Conjugation of gold nanoparticles (GNPs) with silibinin may overcome the above challenges along with distinct advantages of biocompatibility, optical properties for monitoring and causation of cytotoxicity in cancer cells. The current study thus aims to develop silibinin conjugated gold nanoparticles (Sb-GNPs) with pH responsive release in the cancer microenvironment, optimizing several parameters for its higher activity and further evaluate the nanoplatform for their efficacy in inducing cell death *in vitro* against A549 lung cancer cells. GNPs was synthesized using trisodium citrate dihydrate as the reducing agent and further used for the conjugation of silibinin. The synthesized GNPs were found to be monodispersed and spherical in shape. The silibinin was successfully conjugated with gold nanoparticles and long-term stability of GNPs and Sb-GNPs nanoconjugates in suspension phase was confirmed by FTIR and DLS. Anticancer properties of Sb-GNPs were confirmed by different assay using MTT, Trypan blue dye exclusion assay and cell cycle analysis assay. After conjugation of silibinin with GNPs, the efficacy of silibinin increased 4–5 times in killing the cancer cells. This is the first report on using silibinin gold nanoconjugate system for lung cancer therapy with promising future applications.

## Introduction

Despite breakthroughs in cancer understanding, treatment techniques, and novel treatments, cancer patient survival rates have slightly improved. Chemotherapy, immunotherapy, and radiotherapy alone are ineffective cancer treatments. New cancer survival and mortality control techniques are needed (Da Silva et al., 2016; Dai et al., 2017; Pucci et al., 2019; Mahdinloo et al., 2020; Bagherifar et al., 2021). As an alternative or complementary to traditional chemo or radiotherapy, naturally occurring phytochemicals (such as flavonoids, isoflavones, and lignans) with antioxidant or hormone-like actions are being explored for cancer (Ghosh et al., 2021a; Ahmad et al., 2021; Aboushanab et al., 2022; Lim & Park, 2022). These cancer-suppressing phytochemicals act mainly by affecting the signaling molecules that have either been abnormally activated or silenced. These signaling molecules, which are usually kinases, are responsible for activating genes that regulate cell growth, differentiation, and apoptosis (Melim et al., 2022). In order to minimize the chemotherapeutic induced drug toxicities and associated drug resistance in lung cancers, the use of nutraceuticals such as silibinin has taken precedence in recent times. Silibinin is a flavonolignan obtained from the silymarin extract of *Silybum marianum* (milk thistle) plant often explored historically for liver disorders as well as cancers (Abenavoli et al., 2018; Verdura et al., 2021). In some *in-vivo* studies, silibinin has been reported to inhibit angiogenesis, cell proliferation, drug resistance and metastasis in lung cancer models (Mateen et al., 2013). The anti-cancer activity of silibinin is attributed to the suppression of the angiogenic factor VEGF (Vascular Angiogenic Growth Factor) and targeting of multiple cytokines such as IL-1β, TNF-α and IFNγ (Singh et al., 2006; Tyagi et al., 2009). Moreover, transcription factor STAT3 is known to be the intrinsic target of silibinin in primary lung cancers. When activated by the phytochemical, silibinin lowers the levels of VEGF regulators such as cyclooxygenase-2 (COX2) and inducible nitric oxide synthase (iNOS) (Singh et al., 2006; Verdura et al., 2021). Additionally, silibinin is also reported to increase drug sensitivity in drug-resistant lung cancers with acquired mutations in ALK-TKI and EGFR receptors (Singh et al., 2006).

The delivery of any therapeutic molecule comes with a series of challenges. Rapid renal clearance, difficulty in crossing the cell membrane to the cytosol, relatively larger sizes, poor stability, reduced systemic delivery and uneven release profiles are major challenges of using phytochemical molecules without any transfection agents. In terms of pharmacokinetics, only 23%–47% of orally administered silibinin is absorbed from the gastrointestinal tract and is metabolized by cytochrome class of enzymes (Jančová et al., 2007; Ahmad et al., 2021). Conjugation of phytochemical molecules with nanoparticles helps in overcoming the above challenges along with distinct advantages such as desired penetration, controlled release, optimal distribution and specificity (Ghosh et al., 2021b; Srivastava et al., 2021; Melim et al., 2022; Nikdouz et al., 2022). Several nanoplatforms such as those based on glycyrrhizic acid, PVP PEG, PLGA, chitosan, carbon nanotubes, lipid, and magnetic nanoparticles have been explored for conjugation with silibinin (Takke & Shende, 2019). The state-of-the-art has fewer reports on silibinin conjugated metal nanoparticles which could be a nascent, promising area of research with advantageous properties. In this respect, gold (Au) nanoparticles find several medicinal uses because of their inherent biocompatibility (being a non-reactive element), optical properties (for easy detection) and advantagous surface functionalization. Additionally, Au nanoparticles are reported to possess antibacterial and anticancer properties caused by oxidative stress as well as cell DNA damage (Aljarba et al., 2022).

Lung cancer, also known as bronchogenic carcinoma, is a condition characterized by uncontrolled cell development in the lungs. It is the leading cause of cancer-related deaths. Usually, primary lung cancers are carcinomas and develop within the wall or epithelial cells of the bronchial tree (Lemjabbar-Alaoui et al., 2015). Cancers can also arise from the lung’s pleura, mesothelioma, or sometimes from supporting tissues such as blood vessels (Rao & Wei, 2022). Lung cancer is one of the most dangerous and difficult to treat malignancies because it tends to spread early in its course. While lung cancer can spread to any organ in the body, it most commonly spreads to the adrenal glands, liver, brain, and bone (Wang et al., 2022). Various other types of tumours can also metastasize to the lungs. The survival rate in the case of lung cancer is relatively low, although if detected at an early stage, the 5-year survival rate increases to 5–10% through chemotherapy (Pirker & Zhou, 2022). Lung cancer is classified as NSCLC (non-small cell lung carcinoma) or SCLC (small cell lung carcinoma). NSCLC is more common than SCLC but responds less well to chemotherapy. There are three main subtypes of NSCLC, namely squamous cell lung carcinoma, adenocarcinoma and large cell carcinoma or undifferentiated carcinoma. Small cell lung carcinoma (SCLC) is strongly related to smoking. It often originates in the primary and secondary bronchi and proliferates to form large tumors that spread to other parts (Lemjabbar-Alaoui et al., 2015; Pirker & Zhou, 2022). Initial response to chemotherapy is good, but metastasis is high and results in a worse prognosis. Other lung cancers include pleuropulmonary blastoma, carcinoid tumor and metastatic tumors. Mutations that cause the uncontrolled growth of cancer cells are either inherited or acquired. Often, between exposure to a carcinogen and the appearance of cancer, a latency period of years or decades might exist. Mutations in the *K-ras* proto-oncogene are responsible for 10–30% of lung adenocarcinomas (Aviel-Ronen et al., 2006; Herbst et al., 2008; Ghimessy et al., 2020; Tian et al., 2022).

The anticancer activity of silibinin gold nanoconjugate is an unexplored but promising area of research. Conjugates of silibinin and Au nanoparticles are expected to result in a two-way action against persistent tumours. NPs can co-load multiple components for simultaneous administration, shield payloads from degradation and premature release, and passively or actively target tumours through increased permeability and retention or surface modification with ligands (Duan et al., 2019). In the current study, we reported the conjugation of gold nanoparticles with silibinin with pH-responsive release, optimizing several parameters for its increased activity and have further explored their efficacy in inducing cell-death *in-vitro* against A549 lung cancer cells. Collectively, we proposed that such a nanoparticle-mediated system provides a natural product-based anticancer therapeutic platform with promising biomedical applications.

## Material and methods

Tetrachloroauric (III) acid (HAuCl_4_.3H_2_O), trisodium citrate dihydrate (C_6_H_5_Na_3_O_7_. 2H_2_O) were procured from SRL, Mumbai, India. Silibinin, DMSO, RPMI (Roswell Park Memorial Institute medium), fetal bovine serum, 1X trypsin-EDTA solution, MTT powder, were purchased from Sigma Aldrich Chemicals Pvt. Ltd. Human lung adenocarcinoma cell line A549 was obtained from National Centre for Cell Science, Pune (India).

### Preparation of stock solutions of gold salt, trisodium citrate dihydrate and silibinin

Tetrachloroauric acid (1 gm) was dissolved in 50 ml of autoclaved MilliQ water to yield a final concentration of 50.78 mM stock solution of tetrachloroauric acid. Trisodium citrate dihydrate (2.94 gm) was dissolved in 200 ml of autoclaved MilliQ water to yield a final concentration of 50 mM stock solution. The silibinin powder (4.824 mg) was dissolved in 10 ml ethanol (100%) to yield a final concentration of 1 mM stock solution.

### Synthesis of gold nanoparticles

Gold nanoparticles were synthesized by the standard defined method of Turkevich and Frens with slight modifications, where citrate is used as a reducing agent at 100°C (Wuithschick et al., 2015). For the synthesis of gold nanoparticles, the reduction of tetrachloroauric acid solution was carried out by mixing of tetrachloroauric acid (0.5 mM) and trisodium citrate (2.5 mM) and by heating the reaction solution with continuous stirring. Several reaction parameters, including tetrachloroauric acid concentration, trisodium citrate concentration, temperature, and stirring speed were optimised to achieve controlled shape and size of GNPs. Synthesis of GNPs was monitored by using UV-Visible spectroscopy.

### Optimization of concentration of tetrachloroauric acid for GNPs synthesis

Keeping all the other reaction parameters constant, different concentration of tetrachloroauric acid ranging from 0.1 mM to 1.00 mM was taken for the synthesis of GNPs. The reaction was carried out by mixing the tetrachloride with trisodium citrate to provide reducing environment followed by its incubation at 100°C for 30 min to obtain the GNPS. UV-Visible spectra of synthesized GNPs colloidal solution was taken in the range of 200 nm – 900 nm.

### Optimization of concentration of trisodium citrate dihydrate for GNPs synthesis

A range of concentrations of trisodium citrate dihydrate were taken for the optimizing the concentration for the synthesis of GNPs, while keeping all the other reaction parameters constant. The concentrations of trisodium citrate dihydrate ranges from 1.5 mM to 20 mM. A fixed optimized concentration of tetrachloauric acid was mixed with these different concentrations of trisodium citrate dihydrate for the synthesis of Gold NPs at 100°C for 30 min. UV-Visible spectra of Gold NPs colloidal solution was taken to determine the required optimum concentration of trisodium citrate dihydrate for nanoparticles synthesis.

### Optimization of temperature for the GNPs synthesis

Keeping other reaction parameters constatnt, the synthesis of GNPs were carried out at different temperatres (30°C, 45°C, 60°C, 90°C, 105°C and 120°C). A fixed optimized concentration of tetrachloroauric acid and trisodium citrate dihydrate was mixed, and the reaction was done at these different temperatures for 30 min to synthesized GNPs. UV-Visible spectra of synthesized GNPs colloidal solution was taken to determine the optimum temperature.

### Optimization of reaction time for the GNPs synthesis

A range of different time periods were taken for the GNPs synthesis while keeping all the others parameters constant including 1 min, 5 min, 10 min, 20 min, 30 min, 40 min, 50 min, and 60 min respectively. A fixed optimized concentration of tetrachloroauric acid and trisodium citrate dihydrate was mixed, and the reaction was done for the above mentioned time periods at the optimized temperature for the Gold NPs synthesis. UV-Visible spectra of Gold NPs colloidal solution was taken to determine the optimum time of incubation required for the synthesis of GNPS.

### Optimization of stirring speed for the GNPs synthesis

In an attempt to optimize the stirring speed of incubation, the synthesis of GNPs were carried out at 300 rpm, 400 rpm, 500 rpm, 600 rpm, and 700 rpm respectively keeping other parameters constant. After 30 min of incubation, UV-Visible spectra of GNPS colloidal solution were taken to determine the optimum time period required for its synthesis.

### Purification of GNPs

GNPs were purified by centrifuging a prepared sample of colloidal solution for 30 min at 10,000 rpm followed by washing the pellet 2–3 times with double distilled water. The colloidal GNPs solution was centrifuged until a pellet containing clear solution was obtained. The pellet was further dried at 37°C. The pellet was then scraped with a spatula and grounded with a mortar and pestle to produce a fine powder of GNPs for further experiment.

### Conjugation of silibinin with GNPs using physical adsorption process to form silibinin-gold nanoparticles (Sb-GNPs) nanoconjugates

The stock solution of Gold NPs (1 mg/ml) was prepared from the purified powder in double distilled water. This solution was sonicated for an hour at room temperature to obtain the uniform and homogeneous colloidal solution of nanoparticles. Freshly prepared silibinin solution (concentration ranges from 5µM to 100 µM) was then added to GNPs solution and mixed by continuous stirring at 500 rpm for 1 h at 4°C. This process ensured the conjugation of silibinin over the surface of gold nanoparticles. Excess silibinin was removed by centrifuging the sample at 10,000 rpm for 10 min and discarding the supernatant. The pellet of Sb-GNPs nanoconjugates was washed 2–3 times with double distilled water and used for further experiment.

The optimization of silibinin concentration was done by making a saturation curve of silibinin. For this optimization, a range of concentrations of silibinin were taken to be conjugated with GNPs. The different concentrations were 5 μM, 10 μM, 15 μM, 25 μM, 50 μM, 75 μM, and 100 μM, respectively. During these conjugation reactions, the concentration of GNPs as well as other important reaction parameters were kept constant. Further, a saturation curve of silibinin conjugated with gold nanoparticles at different concentrations of silibinin was plotted by taking the absorbance at 288 nm and 528 nm respectively. The amount of unconjugated silibinin in the supernatant of nanoconjugates and the percentage of silibinin binding in nanoconjugates was calculated using the standard curve.

### Characterization

Absorption spectra of the GNPs and nanoconjugates were measured by UV-Visible spectroscopy. The size, shape and morphology of GNPs and nanoconjugates were examined using transmission electron microscopy (TEM), JEOL 2100F microscope operated at 100 kV. Energy-dispersive X-ray analysis was also done using this instrument. Samples for TEM and EDAX were prepared by drop coating purified GNPs onto carbon-coated copper TEM grids. DLS measurements were used to determine the Zeta potential and polydispersity index of the GNPs and nanoconjugates were carried out using the spectroscatterer RiNA, GmbH class3B. The 1 mg powder was dispersed in distilled H_2_O to produce a suitable scattering intensity and values were obtained at an angle of 90° sin 10 mm diameter cells at 20°C for 10 cycles. Fourier transforms infrared (FTIR) spectroscopy was used to determine the functional groups present in silibinin and nanoconjugates respectively. FTIR spectra of the purified GNPs and nanoconjugates were recorded between 4,000 cm^−1^ and 400 cm^−1^ with a resolution of 4 cm^−1^.

### Release study of silibinin with different pH values

Centrifugation of 200 ml of the nanoconjugates solution at 10,000 rpm for 30 min at 4°C resulted in the collection of red pellet at the bottom of centrifuge tube. The collected pellet was distributed into several fractions. The 100 μl of each fraction was diluted with 900 μl of phosphate buffer solution (PBS), which had pH values of 7.4 (physiological) and 5 (acidic, mimicking tumour microenvironement), respectively. The diluted fraction was incubated at 0 min to 6 h, then centrifuged at 10,000 for 10 min at 4°C and collect the supernatants of respective solutions. Further, the absorbance of each supernatant was measured at 288 nm using a UV-Visible spectrophotometer. Further, obtained results were plotted against the percentage release and corresponding time points.

### *In vitro* evaluation of anticancer efficacy of Sb-GNPs nanoconjugates

A549 cells were grown as adherent monolayer in RPMI 1640 containing 10% FBS and 1% antibiotic (pen-strep) at 37°C, and 95% humidified incubator with 5% CO_2_. Sterile conditions were maintained all the times. Exponentially growing cells (sub confluent cells) were used in the entire set of experiments. For treatment with the drug, 10^5^ cells were seeded in a 60 mm culture dish and incubated at 37°C for 48 h. After 24 h of seeding the cells, the consumed medium was removed from the plates. The cells were treated with different concentrations of the drug suspended in the medium and control was treated with the medium without the drug. Each set of treatments was done in triplicates to minimize experimental error. The medium containing different concentrations of the drug was added to each set of plates and the plates were incubated at 37°C.

### MTT assay

Cells were treated with different concentration of drug. After treatment (24 h and 48 h respectively), media was removed and 100 μl of MTT at the concentration of 5 mg/ml in 1X PBS was added to each well. The 96 well plate was incubated at 37°C in 5% CO_2_ incubator for 4 h. The MTT solution was removed and 100 μl of DMSO was added per well. The wells of the plate were read on an ELISA plate reader at 570 nm wavelength after 10 min of incubation in the dark. The data were recorded using the software package SoftPro Max. The cell viability was represented as percent cell viability (Bahuguna et al., 2017).

### Cell death/viability assay by trypan blue dye exclusion

After desired treatments, the cells in the supernatants were collected from each plate and transferred to the corresponding 15 ml tubes. The attached cells were removed after trypsinization (l ml of trypsin/plate). Once the cells were completely detached, 2 ml of chilled 1x PBS was added to each plate. Cells were flushed out of the plate completely and were transferred to corresponding falcon tubes. The cell suspension was spun at 1,500 rpm for 5 min. The supernatant was discarded slowly. The pellet was washed with 1x PBS and the pellet was resuspended in 1 ml fresh 1x PBS. For viability assay, 100 μl of the homogeneous cell suspension was transferred to a microcentrifuge tube. To this suspension, 10 μl of 0.4% trypan blue dye was added. The suspension was mixed well and left for 5–10 min at room temperature. 10 μl of stained cell suspension was pipetted out of the tube and loaded on each chamber of haemocytometer. The procedure was repeated for each set of samples. The percentage of viable cells was calculated by dividing the number of viable cells by a total number of cells multiplied by 100.

### Biocompatibility of silibinin, GNPs and silibinin conjugated GNPs

Biocompatibility study of any drug is very important before its use as a therapeutic. Therefore, in the present study, the biocompatibility of silibinin, GNPs and silibinin conjugated GNPs (Sb-GNPs) against normal human (HEK293T) cell were investigated with the help of MTT assay.

### Hemolysis assay

The hemolytic activity of GNPs, silibinin, and Sb-GNPs were determined as described in our earlier publication (Mishra et al., 2013b). Fresh RBCs were collected from healthy human and washed (1500 RPM; 10 min) three times in PBS buffer (150 mM NaCl, pH 7.4) and re-suspended in PBS at 2% hematocrit. Aliquots of 100 µl suspension were added to wells of 96-well plate containing different concentrations of GNPs, silibinin and Sb-GNPs respectively. PBS alone taken for obtaining baseline values and Triton X-100 (0.4%) in PBS to get 100% hemolysis. After incubation at 37°C for 3 h, the samples were centrifuged and supernatant was used to determine the hemolytic activity measured in terms of hemoglobin release as monitored by absorbance at 415 nm (Molecular Devices, Sunnyvale, CA, United States). Triton X-100 treated control samples were diluted 10-fold before measuring babsorbance. Further, Base line value was subtracted from each data point. Percent hemolysis expressed with reference to 100% hemolysis value (positive control; Triton X-100 treated RBCs).

### Cell cycle analysis

20,000 cells of lung cancer (A549) were attached in 6 well plates and treated with silibinin conjugated gold nanoparticles. After the termination of experiment, all cells were obtained using trypsinization process and centrifuged at 1,200 rpm followed by their processing and FACS study as per earlier reported method (Singh et al., 2021). The cell suspension was ready using the silibinin conjugated gold nanoparticles treated plates, and centrifuged at 1,500 rpm for 5 min. Further, supernatant of centrifuged sample was discarded, and the pellet was again suspended in 500 µl of mixture of saponin and PI solutions [saponin (0.3%), PI (25 mg/ml), EDTA (0.1 mM), and RNase A (10 mg/ml)]. The process was carried out in dark condition. The data were obtained and processed with the help of Cell Quest software in a FACS Cali bur (Becton Dickinson, United States) system for 10,000 events per sample and analyzed after proper gating, to get the cell percentage in each stage of the cell cycle.

### Statistical analysis

All experiments were conducted three times. Their mean ± SD data were observed for statistical significance, values based on one-way ANOVA software along with Dunnett’s post hoc test (Singh et al., 2021). The statistical mean average values are found p*<0.05, p**<0.01, and p***<0.001, matching those of reported value.

## Results and discussion

**FIGURE 1:**
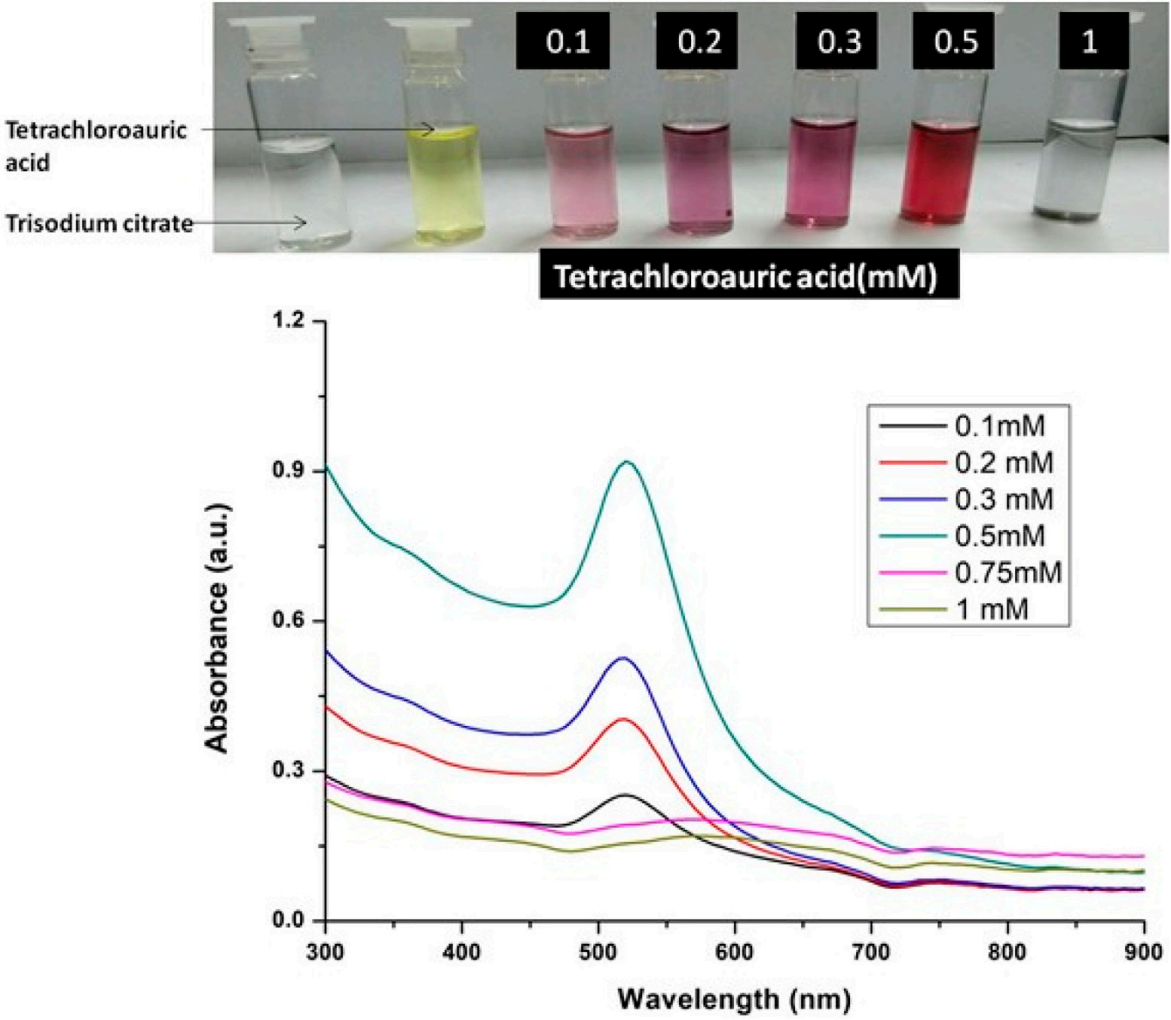
UV-Vis Spectra of Gold nanoparticles solutions synthesised by varying concentrations of tetrachloauric acid. The experiments were done in triplicate and results within each pair differed by < 3.

**FIGURE 2:**
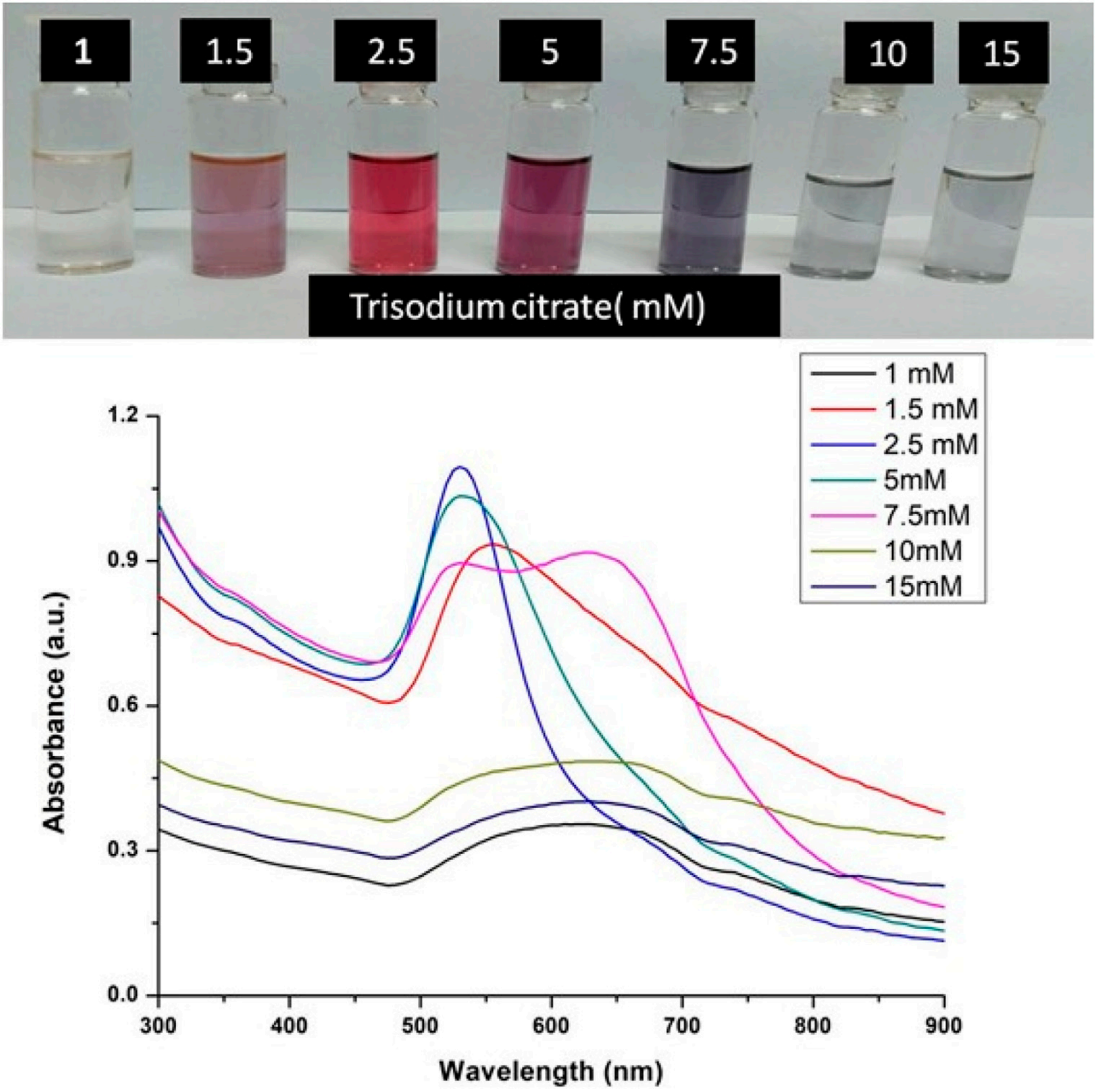
UV-Vis Spectra of Gold nanoparticles solutions synthesised by varying concentrations of trisodium citrate dihydrate. The experiments were done in triplicate and results within each pair differed by < 3.

**FIGURE 3:**
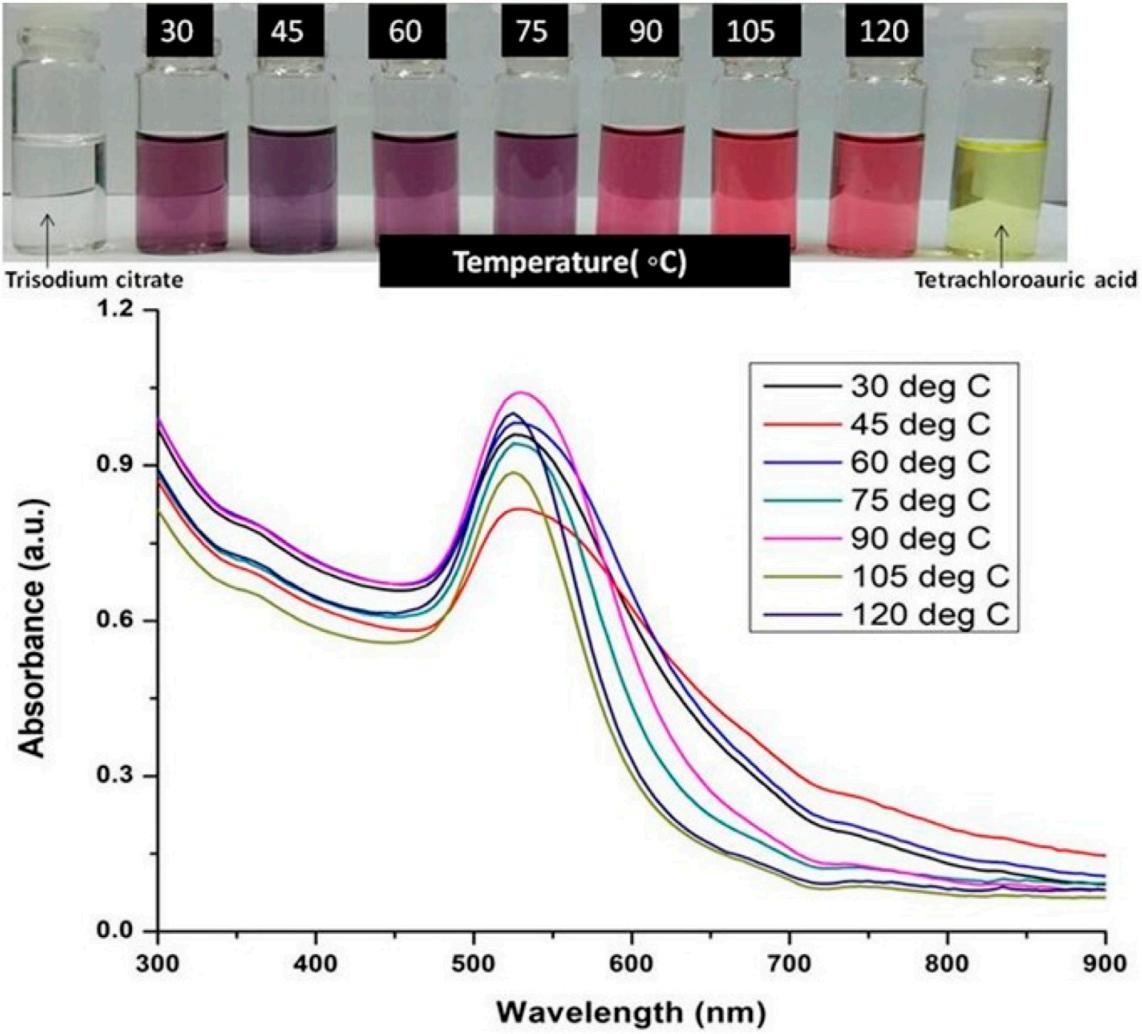
UV-Vis Spectra of Gold nanoparticles solutions synthesised by varying temperature. The experiments were done in triplicate and results within each pair differed by < 3.

**FIGURE 4:**
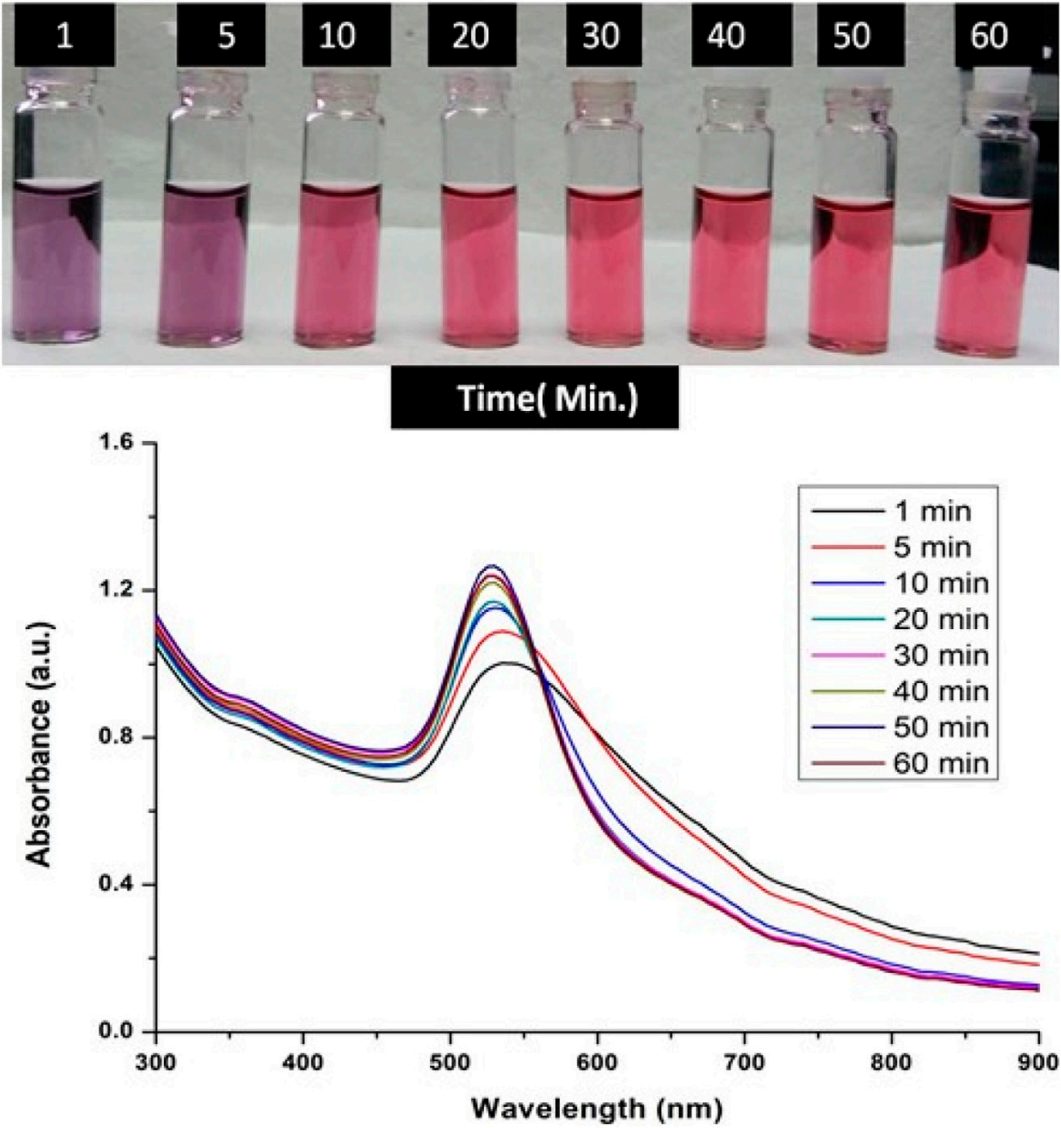
UV-Vis Spectra of Gold nanoparticles solutions synthesised by varying time points of synthesis reaction. The experiments were done in triplicate and results within each pair differed by < 3.

The formation of gold nanoparticles (AuNPs) using trisodium citrate as the reducing agent was observed under visible light due to a change in the color of the solution. The reaction mixture turned brownish-black after 2–3 min of the reaction and then ruby-red due to excitation of surface plasmon vibrations in gold nanoparticles. The appearance of brownish-black colour initially validated the occurrence of a redox reaction whereby Au^3+^ ions are reduced to Au^0^ by trisodium citrate (reducing agent). Gold nanoparticles exhibit a distinct optical feature commonly referred to as localized surface plasmon resonance (LSPR) which is the collective oscillation of electrons in the conduction band of gold nanoparticles in resonance with a specific wavelength of incident light. LSPR of gold nanoparticles resulted a strong absorbance band which can be measured spectroscopically (Figure 1). The LSPR spectrum is dependent both on the size and shape of gold nanoparticles. The peak absorbance wavelength increases with particle diameter and for unevenly shaped particles such as gold nanourchins, the absorbance spectrum shifts significantly into the far-red region of the spectrum when compared to a spherical particle of the same diameter. The absorbance of the sample correlates linearly to the concentration of nanoparticles in the solution. The synthesis of gold nanoparticles at a varying concentration of trisodium citrate dihydrate is shown in Figure 2. This spectrum shows that when the concentration of trisodium citrate dihydrate is 2.5 mM, the sharpest and narrowest peak appears, indicating that nanoparticles of nearly uniform shape and size are synthesized at this concentration. As a result, the optimal concentration of trisodium citrate dihydrate was observed to be 2.5 mM. The synthesis of gold nanoparticles at various temperatures is depicted in Figure 3. This spectrum shows that when gold nanoparticles are synthesized at 90°C, the sharpest and narrowest peak appears, indicating that nanoparticles of nearly uniform shape and size are synthesized at this temperature. As a result, the synthesis reaction’s optimal temperature was determined to be 90°C. Figure 4 depicts the synthesis of gold nanoparticles over time. When comparing the spectra of solutions before and after 30 min, the spectra at 30 min and beyond 30 min displayed a strong and narrow peak. This suggested that the optimum time to make gold nanoparticles was 30 min.

Humans have traditionally used silibinin for various applications, and acute and chronic levels of silibinin administration in animals and humans have shown no harm (Dheeraj et al., 2018). In several pre-clinical cancer models, such as skin, prostate, colorectal, and lung cancer, it has shown significant efficacy in reducing or delaying both tumor initiation and promotion associated events. Because of its widespread use as a popular dietary supplement, biological -tolerance, and low toxicity, silibinin intake has been shown to be beneficial for preventing several disorders. In the given study, we have sought to conjugate silibinin to gold nanoparticles to enhance its efficacy against lung cancer. For this, different concentrations of silibinin were taken for conjugation to gold nanoparticles and maximum binding was observed at 50 μM of silibinin as shown in Table 1. At 50 μM, 80.6% of initial silibinin taken for the conjugation reaction binds to the gold nanoparticles, while only 19.4% remained unconjugated in the reaction mixture. Similar trends in immobilization efficiency with increasing enzyme load have been reported by other researchers (Mishra et al., 2013a; Ahmad et al., 2014b). Based on this observation, for further use in bulk conjugation reactions, 50 μM concentration of silibinin was chosen as the optimum concentration for further experiments.

**TABLE 1:** Table showing the % of conjugated Silibinin to GNPs and % of unbound free Silibinin during conjugation reaction when varying concentrations of silibinin taken for the reaction. The experiments were done in triplicate and results within each pair differed by < 3.

Concentration of silibinin (µM)	Silibinin-GNPs conjugated (%)	Unbound free silibinin (%)
5	54.67	45.33
10	71.77	28.23
15	71.3	28.7
25	75.88	24.12
50	80.6	19.4
75	80.4	19.6
100	77	23.00

**FIGURE 5:**
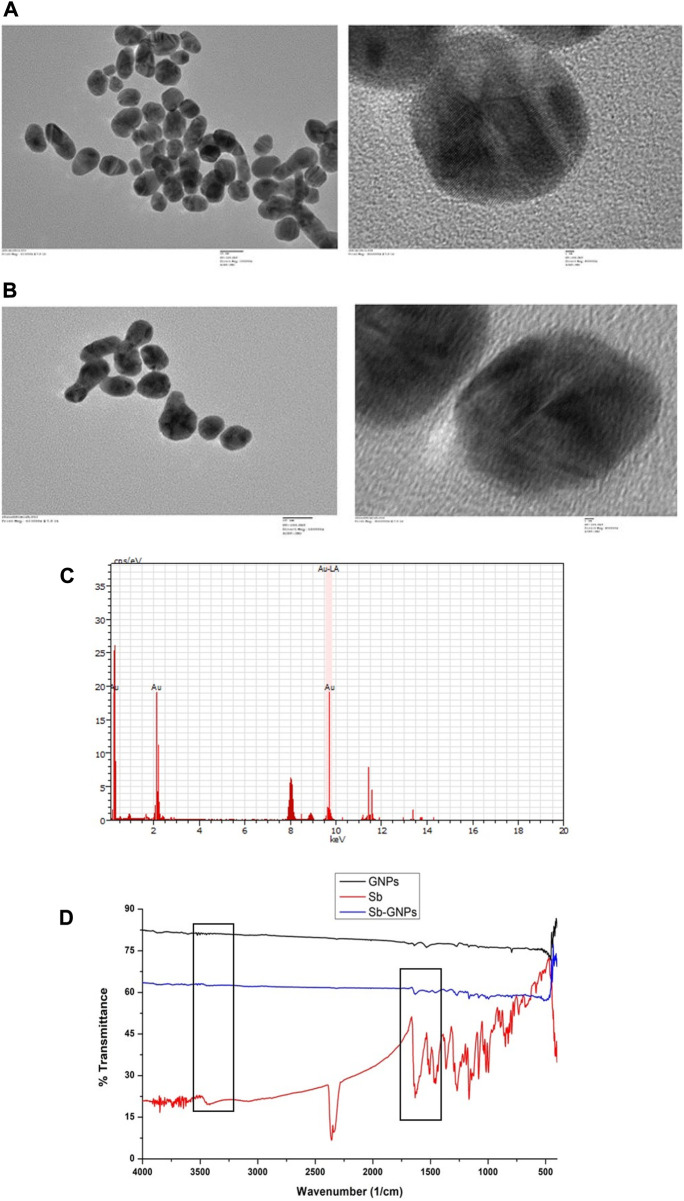
**(A)** Transmission electron microscopy (TEM) images of free, purified gold nanoparticles; **(B)** Transmission electron microscopy (TEM) images of Silibinin conjugated gold nanoparticles; **(C)** Energy dispersive X-ray analysis (EDAX) of gold nanoparticles and Silibinin conjugated gold nanoparticles; **(D)** Fourier transformed infrared spectroscopy (FTIR) spectra of free, purified gold nanoparticles, free Silibinin and Silibinin conjugated gold nanoparticles.

The optical and physical properties of gold nanoparticles were determined by their size (diameter), shape, surface structure, and aggregation state. For functionalizing or conjugating the surface of gold nanoparticles with a biological compound, it is necessary to characterize them to assess the effect of surface functionalization on naked gold nanoparticles. For this, a variety of techniques must be used to characterize both bare and surface-functionalized gold nanoparticles. We have performed TEM to analysed the size, shape and morphology of the GNPs and Silibinin conjugated GNPs. The TEM images of free GNPs and silibinin conjugated GNPs showed nearly monodispersed and spherical shape (Figures 5A,B). Energy-dispersive X-ray spectroscopy (EDAX) is an analytical technique intended for the chemical or elemental analysis of gold nanoparticles. EDAX analysis was performed using TEM. EDAX analysis of silibinin conjugated GNPs showed the presence of gold atom (Figure 5C). The DLS studies were carried out to determine the hydrodynamic radius which reveals that the average diameter was found to be 107 ± 9 nm for GNPs and 163 ± 5 nm for silibinin GNPs nanoconjugates. The increase in size indicates that the silibinin was successfully conjugates with GNPs. The particle size distribution was determined by calculating Polydispersity index (PDI). PDI was found to be 0.3 (GNPs) and 0.5 (Sb-GNPs conjugates). Further, zeta potentials were determined to check the stability of colloidal suspensions and found to be -19.6 mV ± 0.648 (GNPs) and -22.2 mV ± 0.458 mV (Sb-GNPs nanoconjugates), which indicate a long-term stability of gold and Sb-GNPs nanoconjugates in suspension phase. FTIR spectra has indicated sharp peak to understand the possible mechanism of association of silibinin on GNPs (Figure 5D). The IR peak at 1,670 cm^−1^ appeared for stretching vibration of C=O (observed for silibinin) shifting to lower wavenumber in the case of silibinin conjugated gold nanoparticles with reduced intensity thereby indicated the possible interaction of silibinin by its C=O functional group during conjugation to gold nanoparticles. Also, at around 3,400 cm^−1^, peak which showed absorption by -OH functional group of silibinin was observed with reduced intensity in case of silibinin conjugated gold nanoparticles, which indicates the interaction of–OH group of silibinin during conjugation with gold nanoparticles (Ahmad et al., 2015; Sadaf et al., 2020).

**FIGURE 6:**
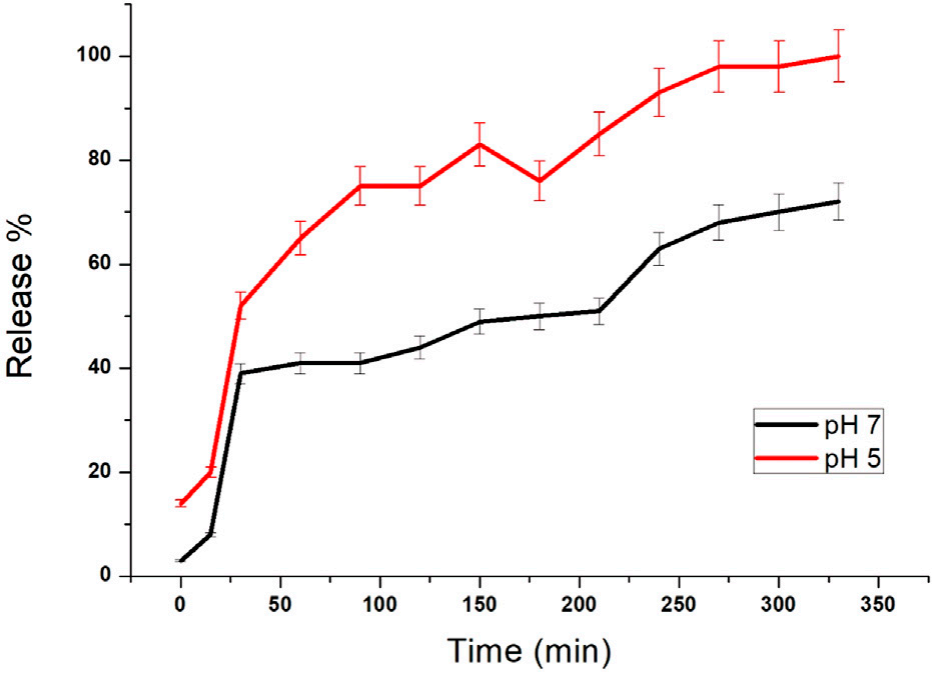
Release study of silibinin from GNPs with different pH values (pH 5 and 7). Each point represents the outcome of a pair of readings, with many points showing errors smaller than the symbols.

**FIGURE 7:**
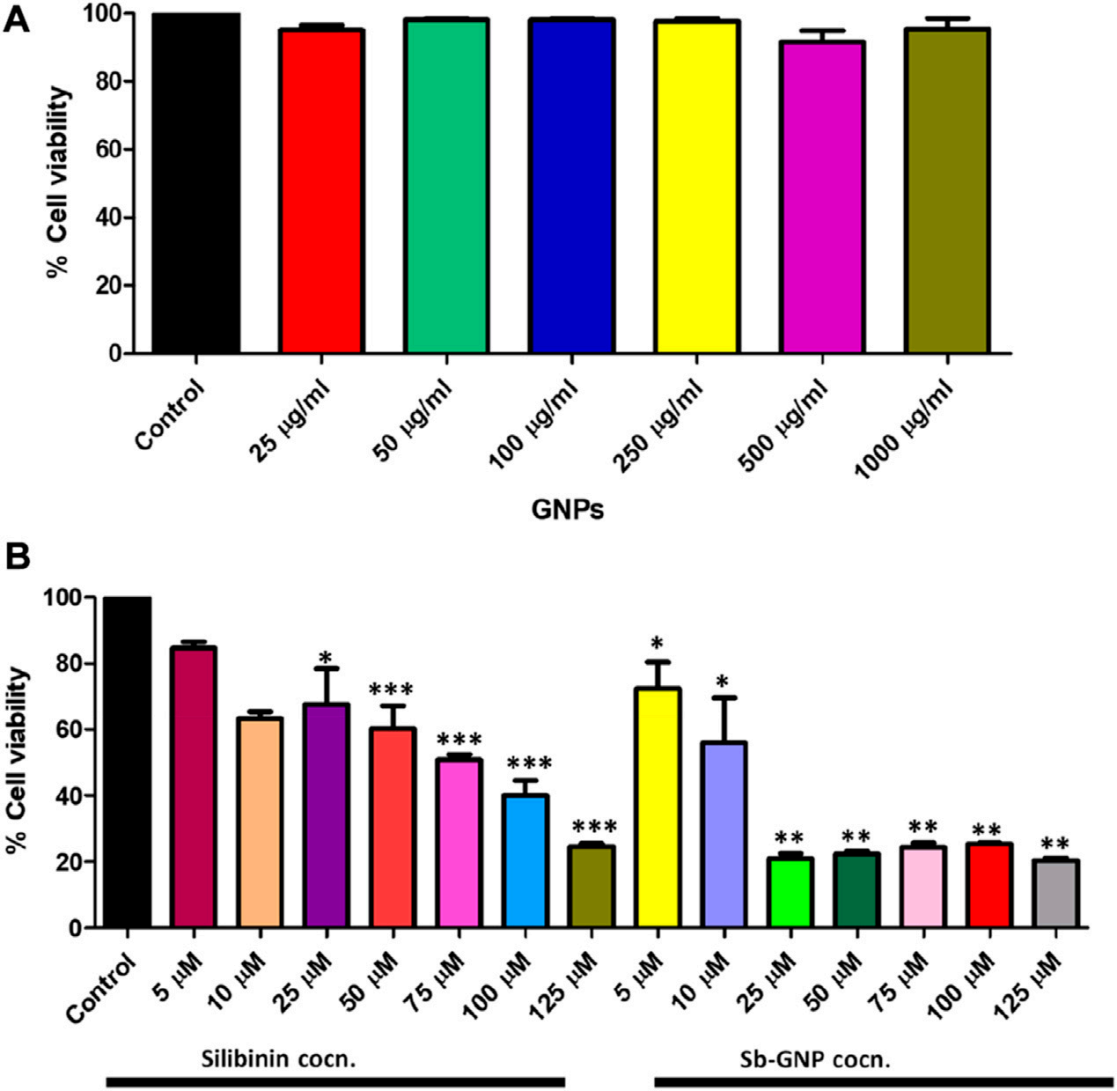
**(A)** *In vitro* anticancer efficacy of free GNPs; **(B)** *In vitro* anticancer efficacy of free Silibinin and Sb-GNPs.

**FIGURE 8:**
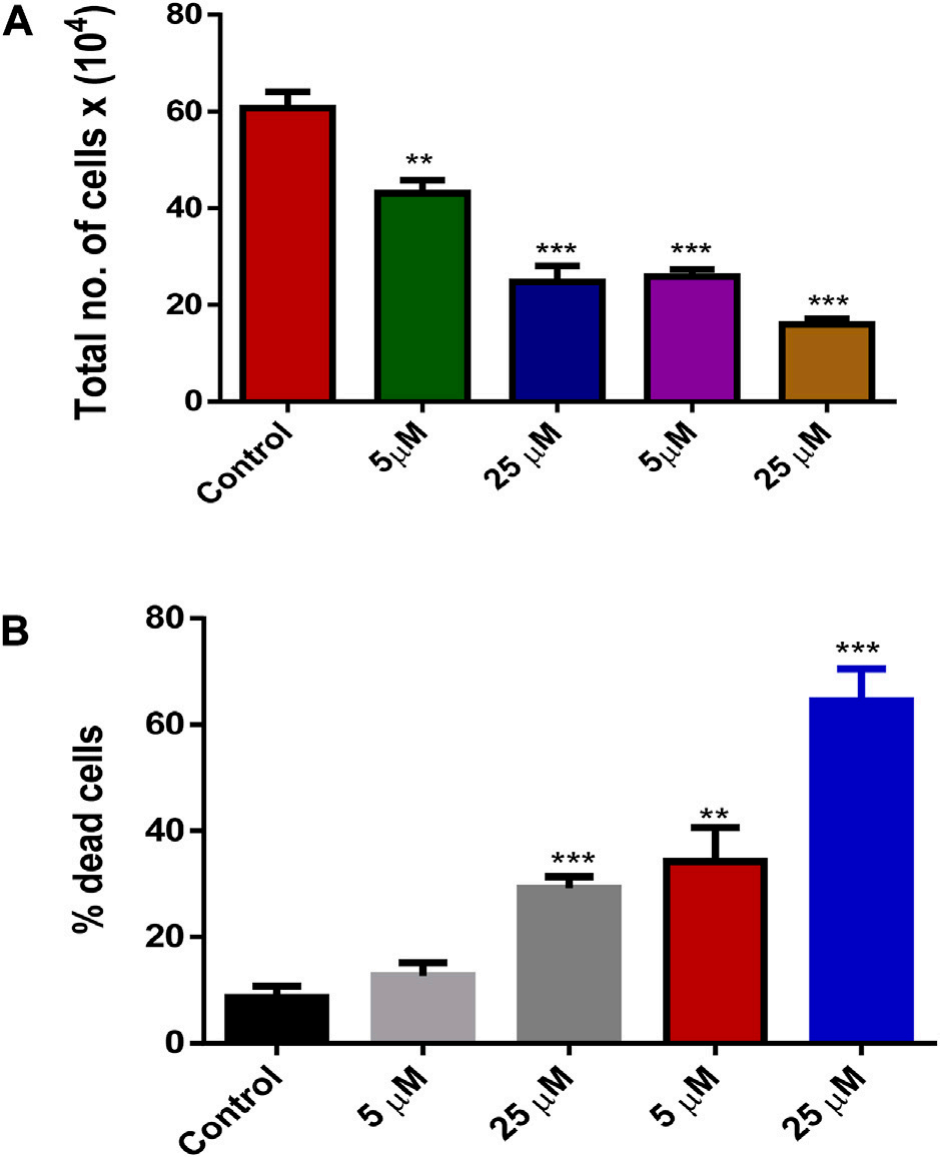
**(A)** Effect of treatment by Silibinin and Silibinin conjugated GNPs on cell growth and viability after 24 h; **(B)** Effect of treatment of silibinin and silibinin conjugated GNPs (Sb-GNPs) on cell death after 24 h.

**FIGURE 9:**
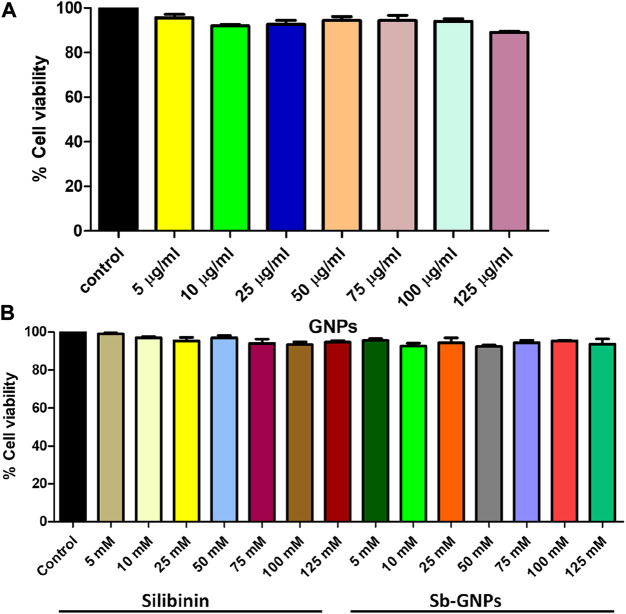
Effect of **(A)** Free GNPs; **(B)** Silibinin and Sb-GNPs against normal cell viability (HEK293T).

**FIGURE 10:**
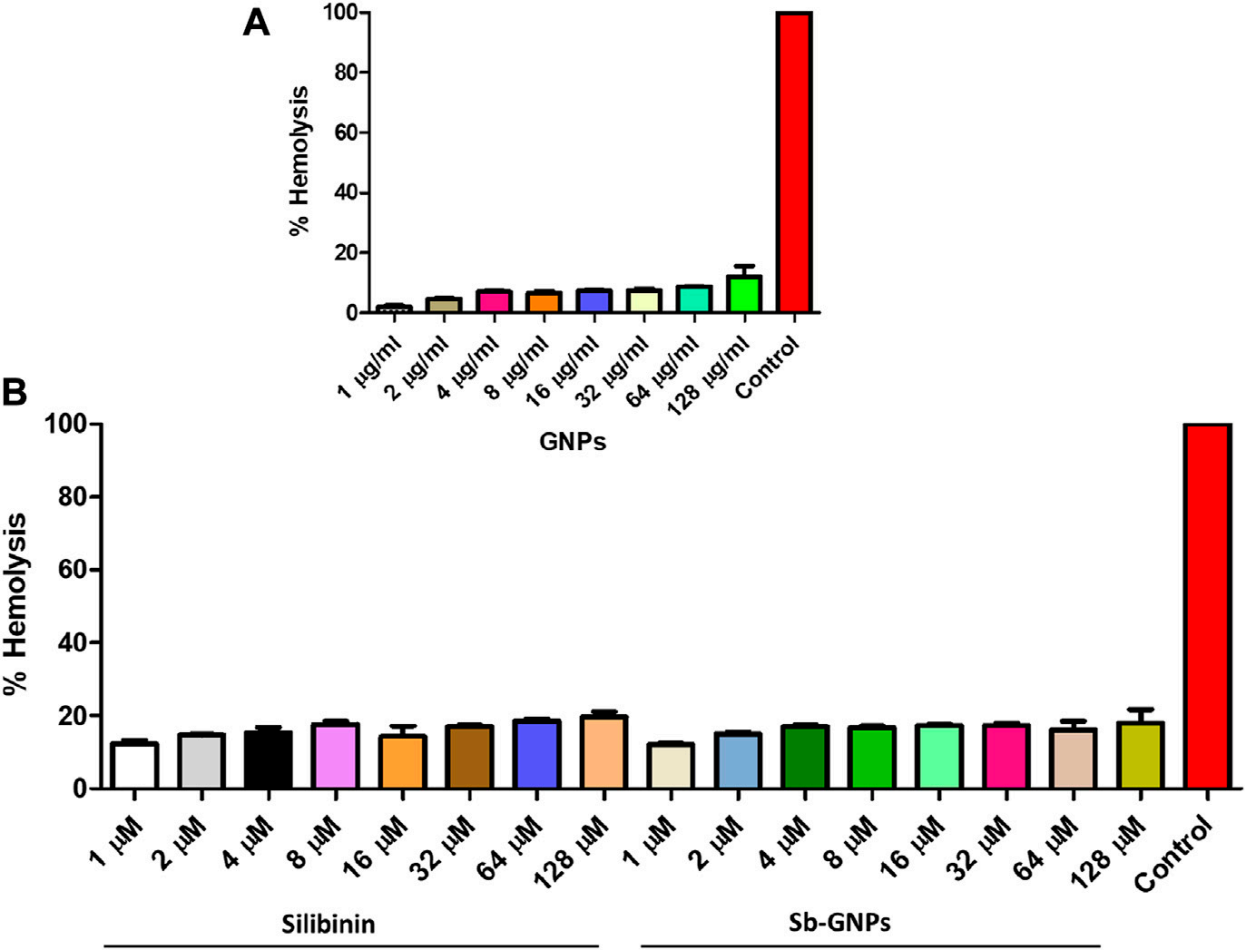
**(A)** Percentage hemolysis of human RBCs caused by GNPs; **(B)** Percentage hemolysis of human RBCs caused by Silibinin and Sb-GNPs. Triton-X-100 (0.4%) was used as positive control.

**FIGURE 11:**
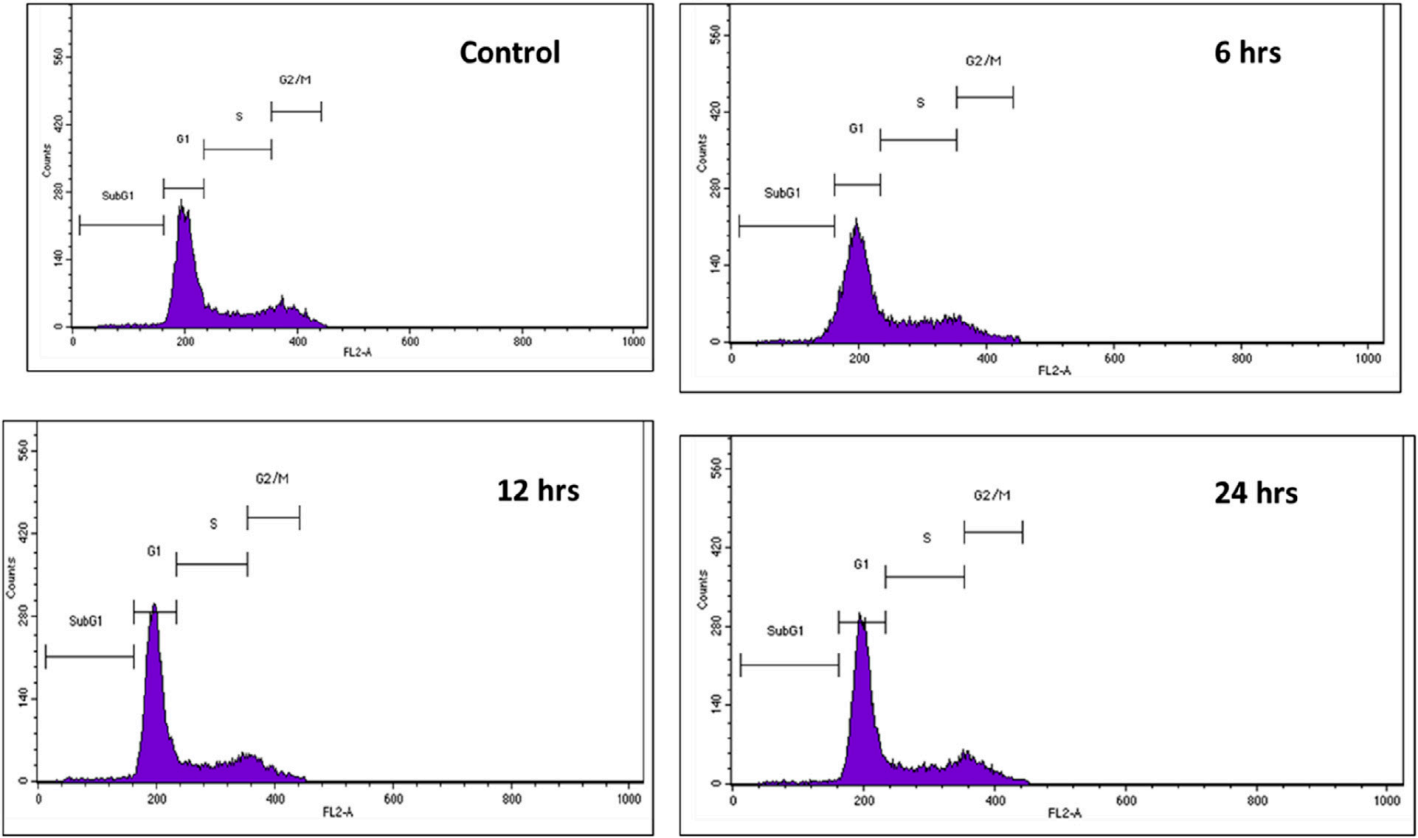
Effect of Silibinin and Sb-GNPs on cell cycle arrest in lung cancer cells.

The release of silibinin in pH 7 (physiological pH) and pH 5 (acidic pH mimicking tumour microenvironment) was monitored by UV-Vis spectroscopy. The result showed that the release of silibinin in acidic pH increased with time from up to 25–200 min and got saturated at 300 min. However, in physiologic pH (pH - 7) lower release of silibinin was observed as compared to release in acidic pH (pH ∼ 5). It is well established that the physiological pH is around 7.4 while that of the tumor microenvironment is in the acidic range. Our results showed that silibinin conjugated gold nanoparticles enhanced the delivery of silibinin to cancer cells (Figure 6). Free gold nanoparticles did not show significant cytotoxicity toward cancer cell lines (Figure 7A). Cell viability of free silibinin and silibinin conjugated gold nanoparticles were tested on lung carcinoma cell line (A549) by performing the MTT assay, and it was found that both have decreased the cell viability. Silibinin conjugated gold nanoparticles showed IC_50_ value at 4.8 μM (w.r.t. Sb concentration) while the IC_50_ value in the case of free silibinin is 24.8 μM Free gold nanoparticles did not show significant cytotoxicity toward cancer cell lines (Figure 7B). IC_50_ value for the formulation was calculated using freely available COMPUSYN software. Trypan blue dye exclusion assay was performed to assess the percentage cell viability after the treatment with silibinin and Sb-GNPs. It was observed that the cell viability decreased more when treated with Sb-GNPs than treatment with free silibinin. The 5 μM dose of conjugated silibinin showed the same decreased in cell viability (approx. 35–40%) as compared to the 25 μM of free silibinin. The results indicates that the low concentration of Sb-GNPs nonoconjugates have achieve the same effect as compared to high concentration of free silibinin. The similar effects can be observed in terms of cell death of the cancer cells. This observation clearly showed that on conjugation with gold nanoparticles, the efficacy of silibinin increased 4–5 times in inhibiting and killing the cancer cells (Figures 8A,B). Further, data of biocompatibility of GNPs, silibinin, Sb-GNPs on normal cell (HEK293) clearly indicate that these compounds are non-toxic in nature. (Figures 9A,B). The investigation of hemolytic activity is important to determine the biocompatibility of GNPs, silibinin and Sb-GNPs with RBCs. Therefore, the hemolytic activity of GNPs at different concentrations (1–128 μg/ml) and for silibinin and Sb-GNPs (1–128 µM) was determined against freshly human RBCs. Figure 10 showed the percentage of hemolysis, which depicts that the hemolysis caused by the GNPs, silibinin and Sb-GNPs is very less as compared with positive control (Triton X100). Thus, the result of the present study indicating the safety of these nanoconjugates (Sb-GNPs) at low concentrations provides support for further investigations. Simillar studies was done by others (Mishra et al., 2013b; Ahmad et al., 2014a).The effect of Sb-GNPs on cell cycle progression of A549 (lung cancer cells) were studied with IC_50_ concentrations obtained by MTT assay. It was found that the IC_50_ dose treatment of Sb-GNPs arrest the growth of cancer cells in G1 phase (Figure 11). Several studies have explored the preparation of silibinin conjugated inhalable nanoparticles as well as Silibinin-loaded PLGA/PEG NPs but that of gold-conjugated ones are few in comparison (Mogheri et al., 2021; Raval et al., 2021). Thus, the above study provides a promising route to develop formulations using silibinin conjugated metal nanoparticles with novel anticancer applications.

## Conclusion

In the present study, Sibilinin conjugated GNPs was synthesized and further investigated their cytotoxic effect on lung cancer cells. Silibinin is of considerable importance which could help prevent normal tissue cytotoxicity in lung cancer patients. Beyond its hepatoprotective effects, the pharmacological activity of silymarin is being revisited due to growing evidence that suggests potential anti-cancer activity. Various evidence indicates that silibinin is a possible drug candidate for tumor growth inhibition and further its efficacy can be enhanced by using nanoparticles (Liu et al., 2020). GNPs was synthesized using trisodium citrate dihydrate as the reducing agent and further it was used as a matrix for the conjugation of silibinin. The techniques TEM, EDAX, DLS and FTIR were performed to characterize the optical and physical properties of gold nanoparticles and its conjugates. The results indicate that the silibilin was successfully conjugated with monodispersed and spherical shape gold nanoparticles.

After characterization, the anticancer efficacy of silibinin and silibinin conjugated gold nanoparticles was evaluated by *in vitro* assays such as MTT assay and Trypan blue dye exclusion assay. It was found that the enhanced cytotoxic and growth inhibitory effects of Gold-Silibinin nanoconjugates as compared to silibinin alone. This opens up the possibility of developing anti-cancer compositions based on silibinin linked nanoparticles.

## Data Availability

The original contributions presented in the study are included in the article/supplementary material, further inquiries can be directed to the corresponding authors.
